# 

**Published:** 1889-03-09

**Authors:** 


**Notice to Advertisers and Subscribers.**

All Advertisements, Orders for Copies of The Hospital, and Business Communications should be addressed to The Publisher, *not to the Editor*, The Hospital, 38, Old Jewry, E.C.

**To Residents, Secretaries, and Matrons.**

The Editor will be glad to have the Regulations and each Report as issued by Asylums, Hospitals, Convalescent and Nursing Institutions, as well as those of other Charities, and an early copy in duplicate of all Appeals and Festival Notices, etc., addressed to *The Editor, The Lodge, Porchester Square, London, W.* Any superintendent, secretary, matron, or other chief official who regularly sends such information may be registered, on application to the Publisher, to receive free of cost a cover for binding The Hospital in half-yearly volumes.

A Scale of Charges will be found on the first page of the Nursing Supplement.

### Scraps and Gleanings.

Darlington Hospital reports a balance in hand of £258.

The last Sunday in February was kept as Hospital Sunday at Croydon.

There are now twenty Pasteur Institutes, of which seven are in Russia.

Dublin Hospital Sunday Fund for 1888 exceeds that of the previous year by £139.

Cirencester Cottage Hospital treated 98 cases last year. Total expenditure £493.

Notts Throat and Ear Hospital treated 515 patients last year. Total expenditure £68.

It costs £500 to endow a cot in Sheffield Children's Hospital, or an annual subscription of £21.

Mr. Jessop having given Sheffield a hospital, we think Sheffield ought to support it.

The general meeting of University College Hospital subscribers will be held on March 27th.

The new Convalescent Home for Derby, the site of which cost £1,950, will be finished in May.

Camborne should be ashamed of itself, it only contributes £9 8s. to the Miners' Hospital.

The Princess Louise has promised to try and open the Leicester Children's Hospital in May.

Janet Hunter, M.D., has been appointed clinical assistant at the Eastern Hospital, Homerton.

Mr. Pratt has given a piece of ground to Newark Hospital, to be used as a garden for patients and nurses.

Dartmouth Cottage Hospital prides itself on having proved its right to existence by treating 36 patients last year.

Cardiff Infirmary is badly in need of an ophthalmic department and an isolation ward. but it has a deficit of £164.

Over two hundred patients were delivered in the National Lying-In Hospital, Dublin, last year, and not one of them died.

An important item in the income of Buchanan Hospital, Hastings, is the patients' payments. There is nothing like self-help.

Glasgow Samaritan Hospital for Women had a heavy year's work in 1888, and now appeal for £5,000 to enlarge their building.

On February 26th the Mayor of Liverpool presided over a Working Men's Committee of the Hospital Saturday Fund of that town.

The percentage of mortality at the Newcastle Hospital for Sick Children last year was 2·8. The cost per patient was £2 14s. 5d.

The Surgical Appliance Society has decided to abandon the "recommends" system, and adopt the free-and-open system of giving aid.

A correspondent writes to the Belfast papers, asking those who are dissatisfied with the Royal Hospital to formulate their complaints.

Greenock Infirmary reports a decrease of expenditure and an increase of income, and finds itself in the unusual position of possessing a surplus.

Since the Manchester Hospital for Consumption demanded that its patients should pay for their cod liver oil, the patients have been less numerous.

The Edinburgh Sick Children's Hospital treated 685 in-patients last year. Colonel W. L. Bathgate has given £1,000 to endow a ward which bears his name.

It is indeed an enlightened age when the Local Government Board asks the Islington Guardians to reform their infirmary nursing and supply trained nurses for the paupers.

Mr. Carpenter, a Hampstead practitioner, has left £45,000 to be divided between the British Medical Benevolent Fund, the British Home for Incurables, and the Orphan Working Schools.

At the last quarterly meeting of Brompton Consumption Hospital Governors, it was announced that 388 patients had been admitted since November, and 2,128 new out-patients had been treated.

A Zanzibar telegram states that the three captive German Catholic missionaries and the Lady Superior of the Pugu Mission House, who were taken taken captive by the Arabs, have been set at liberty.

The Clothworkers' Company has sent the Marchioness of Dufferin and Ava a second donation of £50 towards the fund in support of the National Association for Supplying Female Medical Aid to the Women of India.

The Working Men's Committee of the Hospital Saturday movement in Hull have added to their members representatives of each hospital, the chairman of the infirmary being elected vice-chairman of the committee.

On February 27th, at the Hotel Metropole, the seventy-sixth anniversary festival dinner of the London Orphan Asylum was held. Lord Knutsford presided. The secretary announced subscriptions amounting to £3,053.

By his will just proved, Mr. Alderman Moscrop, ex-Mayor of Bolton, bequeaths to Bolton Infirmary, £2,000; Wesleyan Home Mission Society, £2,000; Children's Home, Edgworth, £2,000. Alderman Moscrop died on January 1st.

The anniversary festival of the Royal Masonic Benevolent Institution was held at the Freemasons' Tavern last week, the Earl of Euston presiding. The list of subscriptions amounted to a total of £13,055, and there are 25 lists still to come in.

The Southampton Hospital Saturday and Sunday Fund has been apportioned as follows: Royal South Hants Infirmary, £652 16s.; Southampton Dispensary, £81 7s. 6d.; Nurses' Training Institute, £28 3s. 3d.; St. Mary's Cottage Hospital, £45 5s.; Shirley Cottage Hospital, £20 17s.; Homœopathic Dispensary, £6 6s. 9d.; Southampton Maternity Society, £4 17s.

Professor Bardeleben, who attended the late Emperor Frederick during the last days of his illness, celebrated his seventieth birthday last week, in circumstances of much honour. Among the congratulations which reached the Professor were telegrams from Sir Spencer Wells, Sir James Paget, Sir Joseph Lister, Sir William MacCormac, and other notabilities in England.

### Amusements and Relaxation.

### NEW PUZZLE PRIZE SCHEME.

**SECOND QUARTERLY COMPETITION COMMENCED JANUARY 5TH, ENDS MARCH 30TH, 1889.**

1. I rizes will be awarded for the best Original Puzzles in Prose or Verse relating to any of the Social, Political, or Philanthropical topics of th day. Competitors are requested to *strictly* observe this rule, or their contributions will be discredited.

2. Puzzles may take any form which the ingenuity of the competitors suggest. Answers must accompany contributions, and the source of obscure or ambiguous terms be given.

3. No competitor will be allowed to take more than one prize during the Quarter.

4. **Eight Prizes of Standard Books** will be given, the choice of the book to be left to the winner. First, Second, Third, Fourth, and Fifth Prizes for best Original Puzzles, value £1 1s., 15s., 10s. 6d., 7s. 6d., and 5s. each.

5. First, Second, and Third Prize for Solutions of 15s., 10s. 6d., and 5s. each.

N.B.—All contributions must reach the office, 38, Old Jewry, E.C., not later than the *First Post* on *Thursday* in each week.

### PUZZLES FOR SOLUTION.

**Tenth Week.**

No. 21. Double Acrostic.

Two sisters stand,
Who, hand in hand,
Together crave discovery.
Two teachers they
Who potent sway
The heart through ear or eye.

1. How do you like it? It's colour is sad,
A curious mixture, æsthetic drab.
2. The name of a coat by Royalty worn,
The name of a province to loyalty sworn.
3. Drawn up, stretch'd out, will nothing relieve it?
Patience and rubbing, if you will believe it.
4. Resign, submit, be this thy creed.
It is enough, Fate hath decreed.
5. Send help and succour to those lying
In this land of famine dying.

Crocodile.

No. 22. Charade.

My *first* is a fish, revers'd a coin or lay,
Or it may be to rob, so some people say;
My *second* a character in fiction behold,
Cruelly robbed by a parent, so we are told;
*Whole* a subject under discussion we see,
The musician inside will help to find me.

Lady Clara.

**Marks for Original Puzzles.**

| Names. | No. of Puzzles sent in Feb. 28. | No. of Marks Feb. 28. | Totals. | Names. | No. of Puzzles sent in Feb. 28. | No. of Marks Feb 28. | Totals. |
|---|---|---|---|---|---|---|---|
| Cotonopolis... | 1 | 3 | 37 | Prior........... | 3 | 4 | 16 |
| Tinie............ | 3 | 4 | 39 | Lightowlers . | 2 | 3 | 20 |
| Jenny Wren . | — | — | 4 | Twentieth ... | — | — | 3 |
| Patience ...... | 3 | 6 | 46 | Crocodile...... | 1 | 3 | 8 |
| Padre ......... | — | — | 3 | Nabob ......... | 1 | 3 | 8 |

**Answers.**

No. 17. Double Acrostic.

N A T I V E

P L A C I D

A B J E C T

D E P U T Y

D R A G O N

S T A I R S

No. 18. Diamond Puzzle.

I
G N U
F I F E R
D A R L I N G
I N F L U E N Z A
B U R E A U X
C A N D Y
I Z E
A

**Marks for Solution of Puzzles.**

Total Marks.—Flora (0), 8; Mater (1), 5; Scrooge (0), 8; Patience 4; Lady Clara (0), 7; Ruffles (0), 6; Lauriston, (0), 1; Reldas (1), 5; Jenny Wren (2), 4; Condorcet 2; Tinie, 2.

[*The Figures in parentheses are Marks for the week ending Feb. 28th.*]

**Conditions.**

I. Every paper sent in must be clearly written, and one side only must be used. All competitors must mark at the head of their papers the number of their competition.

II. Each paper must be signed by the author with his or her real name and address. A *nom de plu e* may be added if the writer does not desire to be referred to by us by his real name. In the case of all prizewinners, however, the real name and address will be published.

III. All communications relating to prize competitions should be addressed to "The Prize Editor, The Hospital, 38, Old Jewry, London, E.C." Competitors are requested not to include in letters, etc., to the Prize Editor, communications on matters of other business. All letters must be fully prepaid.

IV. The Prize Editor of The Hospital is the sole judge of the competitions, and his decision is in all cases final and without appeal.

V. The Prize Editor reserves the right to publish any paper sent in, whether it receives a prize or not. He does not hold himself responsible for any MSS. sent, nor can he undertake to return rejected competitions.

